# High-Performance Biomemristor Embedded with Graphene Quantum Dots

**DOI:** 10.3390/nano13233021

**Published:** 2023-11-25

**Authors:** Lu Wang, Jing Yang, Xiafan Zhang, Dianzhong Wen

**Affiliations:** Heilongjiang Provincial Key Laboratory of Micronano Sensitive Devices and Systems, School of Electronic Engineering, Heilongjiang University, Harbin 150080, China

**Keywords:** starch, graphene quantum dots, PMMA layer, resistive memory

## Abstract

By doping a dielectric layer material and improving the device’s structure, the electrical characteristics of a memristor can be effectively adjusted, and its application field can be expanded. In this study, graphene quantum dots are embedded in the dielectric layer to improve the performance of a starch-based memristor, and the PMMA layer is introduced into the upper and lower interfaces of the dielectric layer. The experimental results show that the switching current ratio of the Al/starch: GQDs/ITO device was 10^2^ times higher than that of the Al/starch/ITO device. However, the switching current ratio of the Al/starch: GQDs/ITO device was further increased, and the set voltage was reduced (−0.75 V) after the introduction of the PMMA layer. The introduction of GQDs and PMMA layers can regulate the formation process of conductive filaments in the device and significantly improve the electrical performance of the memristor.

## 1. Introduction

Traditional computers are based on the Von Neumann architecture, which means that data need to be stored in memory before being transferred to the central processor for processing. However, the shrinking transistor size over the past decades has reached its physical limit, and device optimization alone cannot effectively improve the performance of the central processor. In addition, the frequent data transfers between the processor and the memory also cause huge energy consumption. Therefore, how to solve the Von Neumann bottleneck and improve the data processing capability of computers has become a key issue in the field of information science [1]. Currently, the main approaches are to increase data storage capacity and to develop new computational storage architectures. Novel memories are the key to solving this problem, including reducing the manufacturing cost of memories and increasing their storage density. Resistive random access memory (RRAM) is a type of non-volatile memory that utilizes the resistance change of certain thin film materials under the action of an electric field to achieve information storage. It has the advantages of simple structure, compatibility with the CMOS process, easy integration, fast operation speed, and low power consumption [2,3,4,5,6,7], and, therefore, is attracting much attention in the era of big data. In conclusion, the development of new microelectronic devices is the key to solving the Von Neumann bottleneck and enhancing the data processing capability of computers, and RRAM is one of the new types of nonvolatile memories with the most potential for development.

Up to now, a variety of materials, including organic materials and metal oxides, have been found to possess memory properties. Elemental doping of the dielectric layer and oxide/metal interface modulation are the two main ways to control the formation/breakdown of conductive filaments [8]. For the former, doping with metal nanoparticles [9,10,11], carbon nanomaterials [12,13,14,15,16], quantum dots [17,18,19], etc., is usually used to change the defect energy levels of the dielectric layer. The ITO/DNA:Pbs/metal devices fabricated by Murgunde et al. exhibit bistable nonvolatile storage with ON/OFF ratios approaching four orders of magnitude, and the devices maintain their operating characteristics and good durability even after many cycles (1000) [20]. Yan et al. prepared Ag/ZHO/GOQDs/ZHO/Pt devices by introducing graphene oxide quantum dots (GOQDs) layers into oxide Zr_0.5_Hf_0.5_O_2_ (ZHO) films. Compared with the devices without GOQDs, the devices with GOQDs layers have lower threshold voltages, more uniform setups and reset voltage distributions, faster switching speeds, and lower power consumption [21]. The flexible resistive switching device with Al/CSQDs-PVP/Pt/PET structure, prepared by Zhang et al., exhibited WORM-type characteristics, with a high ON/OFF current ratio (10^5^), low operating voltage (1.6 V), and good stability and flexibility [22]. Valov found that as the device size decreases, the effect of interfacial effects on the device becomes greater [23]. Hong prepared graphene oxide-based memristors and demonstrated the effect of the roughness of graphene oxide on the formation of conductive filaments [24]. Ke investigated the effect of surface roughness on the characteristics of ZnO random-access memories, and the improvement in the switching voltage and resistance distributions of RRAMs with rough surfaces was attributed to the stable distribution of oxygen atoms [25]. Sarkar embedded a layer of GQDs between polymethylmethacrylate (PMMA) sheets as a charge-trapping layer, and the device had a large memory window. In comparison, the device without GQDs control did not have a memory window, demonstrating that the memory window originated from the GQDs, not the trapped states in the PMMA/Si interface [26]. Although RRAMs based on metal oxides and chalcogenides offer reliable switching performance and are compatible with CMOS processes, the environmental impact of the hazardous metals contained therein cannot be ignored [27], and they require high processing temperatures, which makes them unsuitable for use in plastic substrates for flexible electronics.

As science and technology continue to develop and environmental issues intensify, researchers are increasingly focusing on solutions that are non-polluting, sustainable, and have high storage densities as well as fast read/write speeds. It is widely recognized that natural biomaterials are receiving more and more attention in the manufacturing of electronic devices because of their unique properties [9,28,29]. Biological memristors are not only low-cost, but also environmentally friendly [30,31,32,33], and can mimic biological synaptic functions or be used as non-volatile memories [34,35]. Therefore, these natural biomaterials have great potential for applications in biocompatible and disposable electronic devices. Lee compared the performances of RRAM devices based on starch and starch–chitosan composites, both of which operated at lower voltages with good reliability [36]. Chang prepared Al/AP/ITO devices using apple pectin (AP) as the dielectric layer. The devices exhibited stable switching capability, good retention, uniform current distribution, and a high on/off ratio (10^7^), and the switching behavior of the devices mainly originated from the formation and breakage of the conductive filaments in the AP film [37]. Ghosh studied the preparation of a resistive variable memory using the extract of Aloe vera flower as a dielectric layer, and the device had a switching ratio of 75 at a read voltage of 1.5 V, which enabled multiple switching cycles [38]. Qi used lotus leaves (lotus leaves) as the dielectric layer and prepared Ag/lotus leaves/ITO resistive memories by the spin-plating technique. The device showed obvious switching behavior and had a stable storage time over 1000 s. The results of the study also verified the important role of the conductive filament model in organic switching memories [39]. Although bio-memory devices have many advantages, the stability and reliability of most bio-memory devices are relatively poor. The preparation of bio-memory devices usually requires complex biomaterials processing and nano-preparation techniques, which makes the production cost higher and the preparation process more complicated.

Starch is a natural, edible, absorbable, and environmentally friendly biomaterial. In this paper, Al/starch/ITO devices are prepared based on starch in a simple way, and the effects of dielectric layer doping and interfacial doping on the performance of the devices are investigated separately. The test results show that the switching current ratio of the devices with GQDs doped in the dielectric layer was increased compared to the devices with undoped GQDs. Al/PMMA/starch: GQDs/PMMA/ITO devices prepared by embedding a PMMA layer between the dielectric layer and the upper and lower electrode surfaces have better stability and durability with a reduced voltage setting. The conductive mechanism of the device is then explained, describing the effects of the formation of conductive filaments in devices with this structure. This research provides a pathway for developing new microelectronic devices to solve the von Neumann bottleneck and enhance computer data processing.

## 2. Materials and Methods

Starch is an inexpensive, abundant, degradable, and biocompatible polymer that is used as a solid polymer electrolyte in electrochemical systems. Starch has a granular structure, including amylose and amylopectin, which are composed of amylose polymer and branched polysaccharide, respectively. Potato starch, in particular, contains both tightly bound and loosely bound water molecules. Due to the presence of loosely bound water molecules in the microcrystalline network, it has reasonable ionic conductivity. In this paper, potato starch was used as a medium layer to prepare an Al/starch/ITO memristor. Glycerine (C_3_H_8_O_3_) is a colorless, odorless, sweet, viscous liquid used as a plasticizer in this article. For the experiment, 20 grams of starch was dissolved in 10 mL glycerin solution and stirred at a constant ambient temperature for one hour. After the starch solution was filtered through a filter, it was dripped onto the cleaned ITO-glass and ITO-PET substrates and rotated in a homogenizing machine at 500 rpm for 5 s, and then at 2000 rpm for 20 s. The ITO glass, which had been coated with starch film, was placed in a drying oven and dried at 80 °C for 20 min. A total of 25 (5 rows and 5 columns) circular Al electrodes were vaporized on the dielectric layer using a vacuum vapor deposition technique under the mask plate, and each Al electrode had a diameter of about 1 mm and an area of about 0.785 mm^2^. Then, the devices were annealed at 105 °C for 10 min to complete the preparation of the Al/starch/ITO devices. Al/starch: GQDs/ITO devices were prepared by adding 30 wt% GQD solution (1 mg/mL) into the starch solution and using the starch and GQD composite material as the medium layer. PMMA powder was dissolved in anisole to prepare a PMMA solution with a concentration of 70 mg/mL. The PMMA solution was coated on the clean substrate at a speed of 1000 rpm, and after 20 s, the PMMA layer was obtained by vacuum drying at 80 ℃ for one hour. The composite solution of starch and GQDs was dripped onto the substrate and rotated at 500 rpm for 5 s, then at 2000 rpm for 20 s. The substrate was placed in a drying oven and dried at 80 °C for 20 min. Then, in the same process of preparation of the PMMA layer and vacuum evaporation Al electrodes, the Al/PMMA/starch: GQDs/PMMA/ITO preparation was completed.

The UV–Vis spectra of materials were obtained with an ultraviolet–visible spectrophotometer (UV/VIS, TU-1901) (Beijing Purkinje General Instrument Co., Ltd., Beijing, China). The infrared spectra of the starch films were measured by a Fourier transform infrared spectrometer (Thermo Fisher 1410) (Thermo Fisher, Waltham, MA, USA). The cross sections of the device were characterized by scanning electron microscopy (Hitachi S-3400 N) (Hitachi, Tokyo, Japan), and the electrical characteristics of the prepared device were tested using a semiconductor parameter tester (Keithley 4200) (Keithley, Solon, OH, USA).

## 3. Results

**Figure 1:**
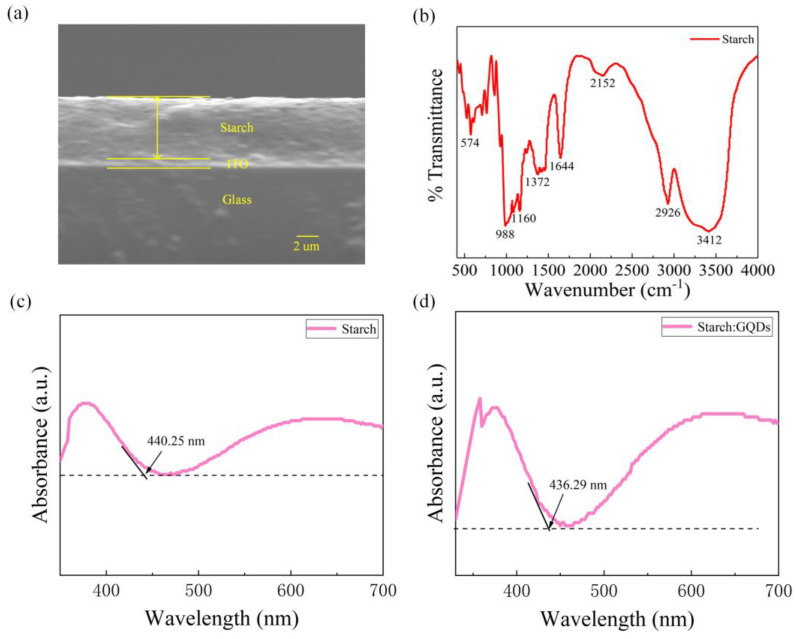
(**a**) SEM images of starch film cross-section; (**b**) Fourier transform infrared spectroscopy of starch film. (**c**,**d**) Ultraviolet visible absorption spectrum: (**c**) starch thin film, (**d**) starch and GQDs composite thin film.

The cross-sectional area of the starch film was observed using a scanning electron microscope (SEM). Figure 1a shows that the starch film, ITO electrode, and glass substrate were sequentially arranged from top to bottom. The thickness of the starch film was approximately 5.86 µm. The Fourier transform infrared (FTIR) spectroscopy test of the starch film is shown in Figure 1b. The peak at 574 cm^−1^ corresponds to the skeletal mode vibration of the starch [40,41]; 988 cm^−1^ corresponds to the asymmetric ring mode of starch; 1160 cm^−1^ corresponds to the C-O stretching vibration and C-C stretching vibration; 1372 cm^−1^ and 1644 cm^−1^ correspond to the asymmetric and symmetric stretching vibration of carboxylate (-COO-Na); and 2926 cm^−1^ corresponds to the antisymmetric stretching vibration of CH_2_. At 3412 cm^−1^, there is a hydroxyl vibration peak that forms hydrogen bonds.

**Figure 2:**
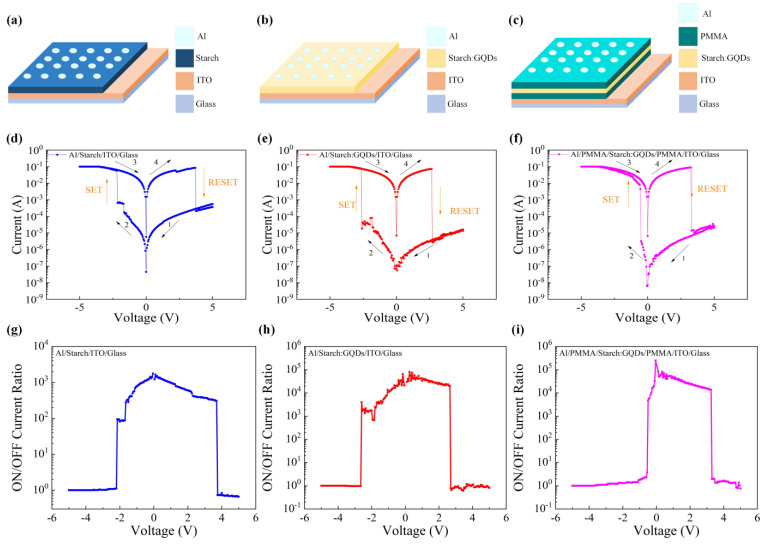
(**a**–**c**) Device structure schematic: (**a**) Al/starch/ITO/glass, (**b**) Al/starch: GQDs/ITO/glass, (**c**) Al/PMMA/starch: GQDs/PMMA/ITO/glass. (**d**–**f**) I–V characteristics curves: (**d**) Al/starch/ITO/glass, (**e**) Al/starch: GQDs/ITO/glass, (**f**) Al/PMMA/starch: GQDs/PMMA/ITO/glass. (**g**–**i**) ON/OFF current ratio: (**g**) Al/starch/ITO/glass, (**h**) Al/starch: GQDs/ITO/glass, (**i**) Al/PMMA/starch: GQDs/PMMA/ITO/glass.

UV-visible absorption spectra of the starch films and starch: The GQDs composite films used in this paper were analyzed using a UV-visible spectrophotometer, as shown in Figure 2c,d. The bandgap width of the material was found according to the formula E_g_ = hc/λ, where Eg is the bandgap width, h is Planck’s constant, c is the speed of photons, and λ is the absorption wavelength. The tangent method showed that the absorption peak edge of the starch film was located at the wavelength of 440.25 nm, and the forbidden bandwidth of the starch was calculated to be 2.82 eV. The absorption peak edge of the starch: GQDs film was located at the wavelength of 436.29 nm, and the forbidden bandwidth of the starch: GQDs was calculated to be 2.84 eV for the same reason.

The structures of the Al/starch/ITO/glass, Al/starch: GQDs/ITO/glass, and Al/PMMA/starch: GQDs/PMMA/ITO/glass devices designed in this article are shown in Figure 2a–c, respectively. The electrical characteristics of these devices were tested at room temperature. During the test, a DC voltage was applied to the Al electrode, the ITO bottom electrode was grounded, and its scanning direction was along the direction labeled 1→2→3→4 in the figure. As shown in Figure 2d, for Al/starch/ITO/glass devices, during the first voltage scan, the device exhibited HRS. When the voltage increased from 0 V to −2.20 V, the negative bias drove the resistance state of the device to transition from the HRS to the LRS. Subsequently, a voltage of opposite polarity was applied, and the voltage increased to 3.75 V, changing the resistance state from LRS to HRS. This device can repeatedly switch between the HRS and LRS by changing the applied bias polarity. The I–V characteristics of the Al/starch: GQDs/ITO/glass devices are shown in Figure 2e. When the voltage increased from 0 V to −2.65 V, the device resistance state transitioned from HRS to LRS; subsequently, a voltage of opposite polarity was applied, and the voltage increased to 2.70 V. The resistance state of the device returns from LRS to HRS. To investigate the influence of the PMMA layer on the electrical characteristics of the devices, electrical tests were conducted on Al/PMMA/starch: GQDs/PMMA/ITO/glass devices, as shown in Figure 2f. The devices exhibited stable bipolar resistance switching behavior. The set voltage and reset voltage were −0.75 V and 3.30 V, respectively, indicating that the set voltage was lower than that of the first two devices. The ON/OFF current ratios of Al/starch/ITO/Glass, Al/starch: GQDs/ITO/Glass, and Al/PMMA/starch: GQDs/PMMA/ITO/Glass devices are shown in Figure 2g–i, respectively. All three devices could be switched between HRS and LRS by changing the applied bias polarity between HRS and LRS repeatedly, where the maximum ON/OFF current ratio of Al/starch/ITO/Glass device was 1.76 × 10^3^, that of Al/starch: GQDs/ITO/Glass device was 7.99 × 10^4^, and that of Al/PMMA/starch: GQDs/PMMA/ITO/Glass devices had a maximum ON/OFF current ratio of 2.42 × 10^5^. We used the same ITO-glass substrate as the Al/PMMA/starch: GQDs/PMMA/ITO/glass device, prepared the PMMA layer and the Al electrode using the same process as before, fabricated the Al/PMMA/ITO device, and measured the electrical properties. Figure S1 shows the I–V characteristics of 40 units for the Al/PMMA/ITO memristors, which shows that the PMMA layer prepared based on this process was a conductor with high resistance.

**Figure 3:**
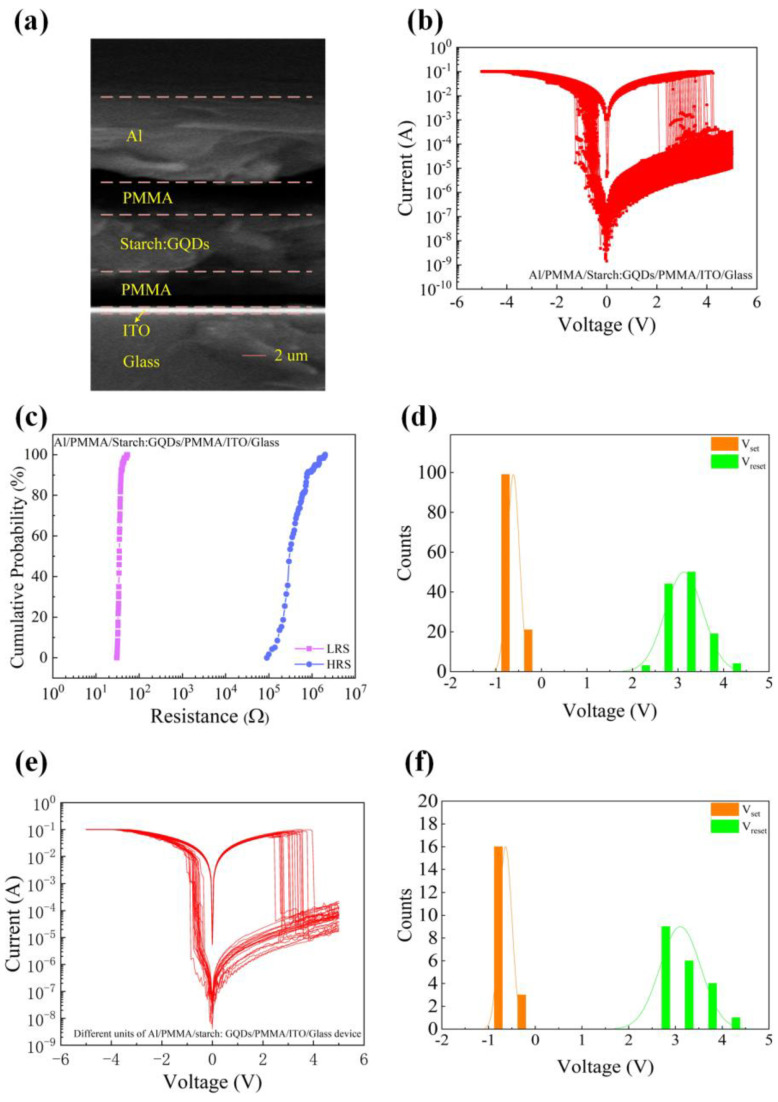
(**a**) SEM image of a cross-section of the Al/PMMA/starch: GQDs/PMMA/ITO/glass specimen. (**b**–**d**) Al/PMMA/starch: GQDs/PMMA/ITO/glass device’s electrical characteristics: (**b**) the I–V characteristics of 120 consecutive cycles of the same memory cell, (**c**) cumulative resistance probability of a component, (**d**) threshold voltage distribution. (**e**,**f**) Different units of Al/PMMA/starch: GQDs/PMMA/ITO/glass device: (**e**) I–V characteristic curves, (**f**) threshold voltage distribution.

The cross-section of the Al/PMMA/starch: GQDs/PMMA/ITO/glass device was observed using a scanning electron microscope. In Figure 3a, the Al electrode, the first PMMA layer, the starch: GQDs film, the second PMMA layer, the ITO electrode, and the glass substrate are shown in order from top to bottom, where the Al electrode has a thickness of 7.37 µm, the first PMMA layer has a thickness of 2.81 µm, the starch: GQDs film has a thickness of 5.39 µm, the second PMMA layer has a thickness of 2.97 µm, and ITO has a thickness of 197 nm. Both PMMA layers are barrier layers, and the starch: GQDs film is the dielectric layer. To demonstrate the stability of the switching behavior of the Al/PMMA/starch: GQDs/PMMA/ITO/Glass devices, the I–V curves of the tested devices over 120 consecutive cycles are shown in Figure 3b. The cumulative probabilities of resistance for 120 consecutive switching cycles of the same memory cell of the Al/PMMA/starch: GQDs/PMMA/ITO/Glass device are shown in Figure 3c, where the device has a coefficient of variation of 0.54 at HRS and 0.10 at LRS. The threshold voltage distributions of the devices were counted as shown in Figure 3d, in which the average value of V_set_ of Al/PMMA/starch: GQDs/PMMA/ITO/Glass devices was −0.62 V with a standard deviation of 0.13, and the average value of V_reset_ was 3.13 V with a standard deviation of 0.42. The experimental results show that the device had good stability and was able to switch between HRS and LRS stably.

Figure 3e shows the I–V characteristic curves of 20 different cells of Al/PMMA/Starch: GQDs/PMMA/ITO/Glass devices under the same test conditions. It is easy to see that there were no obvious differences in the I–V characteristic curves of the 20 cells, and the devices showed good consistency. The threshold voltage distributions of the different cells are shown in Figure 3f. The average value of V_set_ was −0.65 V with a standard deviation of 0.14, and the average value of V_reset_ was 3.11 V with a standard deviation of 0.44.

The experimental results show that the Al/PMMA/Starch: GQDs/PMMA/ITO/Glass device has good reproducibility from device to device.

**Figure 4:**
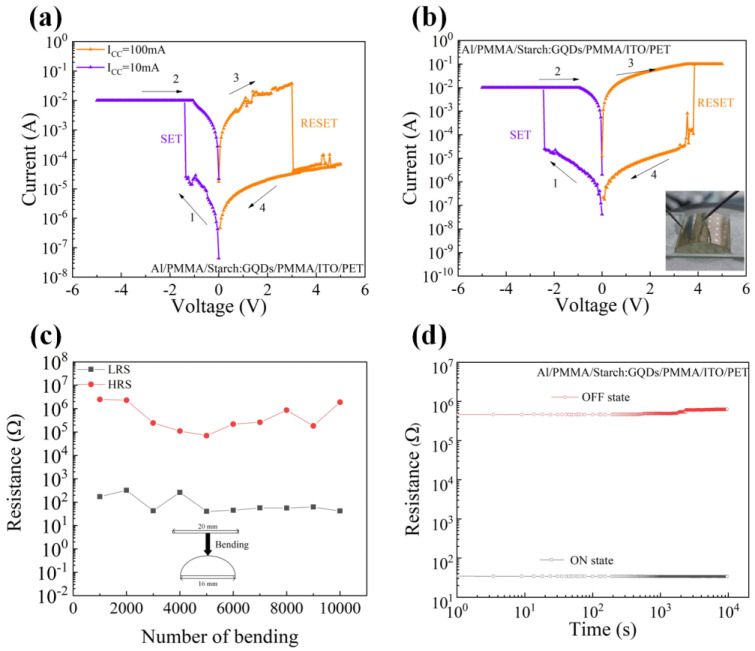
(**a**) I–V characteristic curve of the Al/PMMA/starch: GQDs/PMMA/ITO/PET device; (**b**) I–V characteristic curve under the device bending state; (**c**) R_LRS_ and R_HRS_ after 10^4^ device bends; (**d**) retention time.

The electrical characteristics of the Al/PMMA/starch: GQDs/PMMA/ITO/PET memristors are shown in Figure 4. Firstly, the behavior of the switching resistor in the current-limiting case was explored, as shown in Figure 4a, where I_CC_ = 10 mA was set in the negative voltage region and I_CC_ = 100 mA was set in the positive voltage region, and the voltage scanning direction was 0 V→−5 V→0 V→5 V→0 V. In the negative voltage region, the device current suddenly increased when the voltage increased to −1.40 V, and the device switched from the HRS to the LRS. In the positive voltage region, when the voltage increased to 3.00 V, the current of the device suddenly decreased and the device reverted to HRS. The device was able to switch between high and low resistive states under a continuous voltage sweep. In order to investigate the flexible performance of the Al/PMMA/Starch: GQDs/PMMA/ITO/PET devices, the electrical characteristics of the devices were tested when they were in the bending state. When the flexible Al/PMMA/starch: GQDs/PMMA/ITO/PET devices (substrate size: 20 mm × 20 mm) were gradually bent from the flat state to a diameter of 16 mm, the devices were still able to operate normally. When bent to a diameter of less than 16 mm, the electrical characteristics of the flexible device were affected. The I–V characteristic curves of the Al/PMMA/starch: GQDs/PMMA/ITO/PET devices were tested under bending, as shown in Figure 4b, and the devices still maintained their bipolar resistive switching behavior. In addition, in order to test its stability after bending, we tested the high and low resistance state distributions of the device after 10,000 bending cycles, as shown in Figure 4c. The results show that the high and low resistance states of the device showed no obvious change after 10,000 bending cycles and had strong stability. In order to investigate the stability of the Al/PMMA/starch: GQDs/PMMA/ITO/PET devices, the resistance values of the high and low resistance states of the devices were read at 1 V DC voltage, as shown in Figure 4d, and the devices’ retention times were more than 10^4^ s.

**Figure 5:**
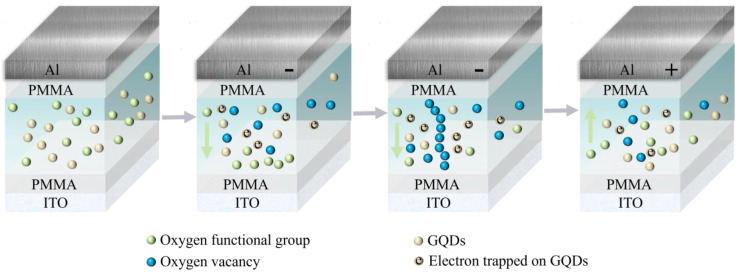
Diagram of the conductive mechanism of Al/PMMA/starch: GQDs/PMMA/ITO.

The conductive mechanism of the Al/PMMA/starch: GQDs/PMMA/ITO device is shown in Figure 5, and the switching behavior of the device may have been due to the formation and breakage of the conductive filaments consisting of oxygen vacancies. The top and bottom electrodes of the device are inert electrodes, which are not involved in the formation of conductive filaments. Whereas starch contains a certain amount of oxygen-containing groups, PMMA also contains oxygen-containing groups. The initial state of the device was HRS, and when a negative voltage was applied to the Al electrode, oxygen ions migrated to the ITO bottom electrode, leaving oxygen vacancies. At the same time, due to the charge trapping effect of GQDs, some of the injected electrons were trapped by GQDs, which introduced more oxygen-containing groups into the dielectric layer and increased the energy band barrier, thus deepening the electron-trapping depth of the composite film, increasing the initial resistance of the device, and improving the ON/OFF current ratio of the device. When the negative voltage was increased to V_SET_, the oxygen vacancy conductive path was formed and the device switched from HRS to LRS. When a positive voltage was applied to the Al electrode, the electrons captured by the GQDs were released and combined with the oxygen vacancies, the conductive filaments were fractured in the local area, and the resistive state changed from LRS to HRS. The PMMA layer had a large bandgap width, which impeded electron movement. Thus, the electrons injected into the starch: GODs layer continued to accumulate and formed a localized electric field, which reduced the randomness of the formation of the conductive filaments, improved the stability, and caused the device’s high-resistance state resistance and the ON/OFF current ratio to increase.

## 4. Conclusions

In this paper, Al/starch/ITO, Al/starch: GQDs/ITO, and Al/PMMA/starch: GQDs/PMMA/ITO memristors were prepared using starch as the main material of the memristors’ dielectric layers. The experimental results show that the maximum ON/OFF current of the Al/starch: GQDs/ITO/Glass device was improved to 7.99 × 10^4^ compared to the Al/starch/ITO device, while the Al/PMMA/Starch: GQDs/PMMA/ITO/Glass memristor, which had a much smaller reset voltage (−0.75 V), had a ON/OFF current of more than 10^5^. A single memory cell can be repetitively switched more than 120 times. The Al/PMMA/starch: GQDs/PMMA/ITO/PET flexible device was successful in terms of the bipolar switching storage method even after bending 10,000 times.

## Data Availability

The datasets used and/or analyzed in the current study are available from the corresponding author upon reasonable request.

## Supplementary Materials

The following supporting information can be downloaded at: https://www.mdpi.com/article/10.3390/nano13233021/s1. Figure S1. I–V characteristics of 40 units for Al/PMMA/ITO memristors. (PDF).
