# Supplementary material for: High-Performance Biomemristor Embedded with Graphene Quantum Dots
Source: Nanomaterials (Basel). 2023 Nov 25;13(23):3021. doi: 10.3390/nano13233021 (PMC10708532; doi:10.3390/nano13233021)
Supplement: Supplementary file 1 [file nanomaterials-13-03021-s001.zip › nanomaterials-2628314-supplementary.pdf]

Supporting Information

High-performance biomemristor embedded with graphene quantum dots

Lu Wang, Jing Yang, Xiafan Zhang and Dianzhong Wen*

School of Electronic Engineering, Heilongjiang University, Harbin, 150080, China

Corresponding Author's email: wanglu@hlju.edu.cn

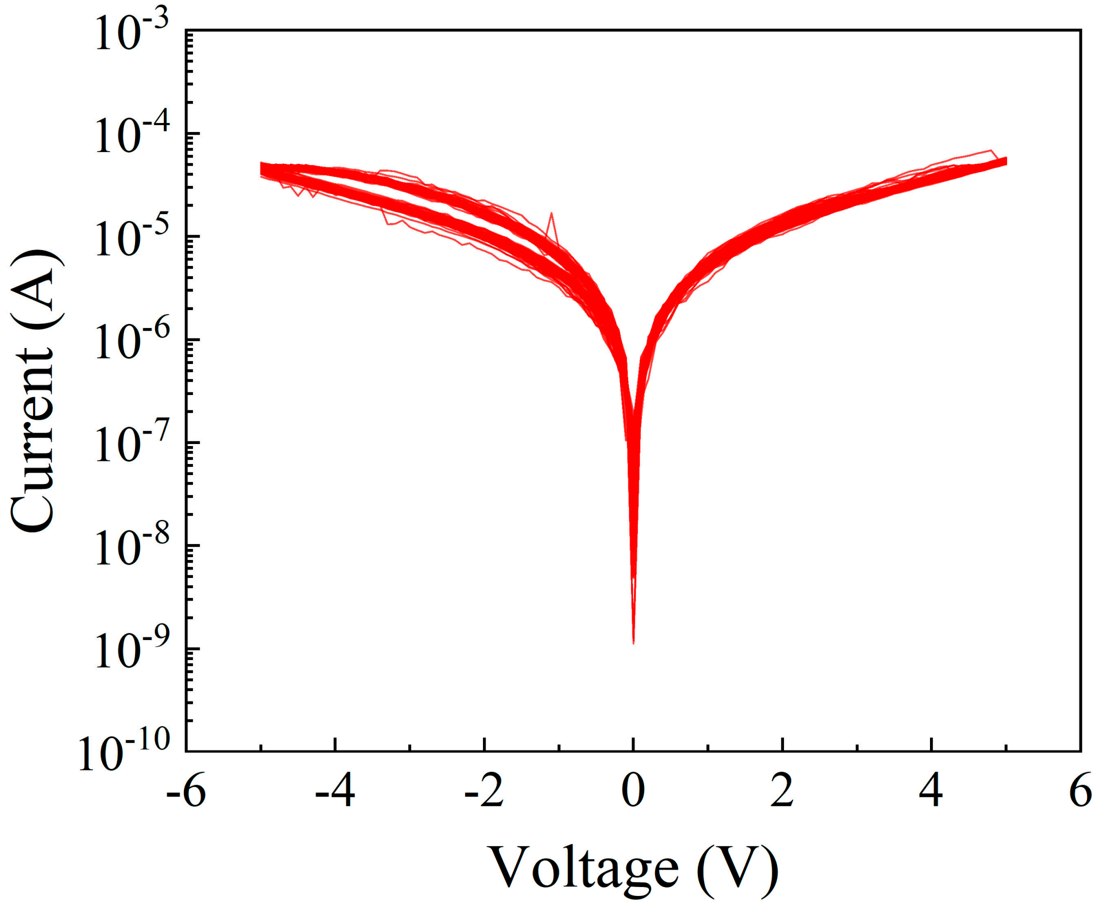

Figure S1. I-V characteristics of 40 units for Al/PMMA/ITO memristors.
